# Effects of 5-Hydroxymethylfurfural on Pubertal Development of Female Wistar Rats

**DOI:** 10.4274/jcrpe.galenos.2019.2019.0080

**Published:** 2020-03-19

**Authors:** Selin Elmaoğulları, Elçin Kadan, Elvan Anadol, Ayris Gökçeoğlu, Semra Çetinkaya, Gül Fatma Yarım, Seyit Ahmet Uçaktürk, Zehra Aycan

**Affiliations:** 1 University of Health Sciences Turkey, Dr. Sami Ulus Children Training and Research Hospital, Clinic of Pediatric Endocrinology, Ankara, Turkey; 2 University of Health Sciences Turkey, Gülhane Training and Research Hospital, Clinic of Pathology, Ankara, Turkey; 3 Gazi University, Laboratory Animal Breeding and Experimental Researches Center, Ankara, Turkey; 4 Ondokuz Mayıs University Faculty of Veterinary Medicine, Department of Biochemistry, Samsun, Turkey; 5 University of Health Sciences Turkey, Ankara Children Diseases Hematology and Oncology Training and Research Hospital, Clinic of Pediatric Endocrinology, Ankara, Turkey

**Keywords:** Hydroxymethylfurfural, puberty, vaginal opening, anti-Müllerian hormone, rat

## Abstract

**Objective:**

5-Hydroxymethylfurfural (HMF) is formed when sugars are heated in the presence of amino acids. HMF is naturally present in many foods. To investigate the toxic effects of HMF on the reproductive system of peripubertal rats.

**Methods:**

In the study, 24 immature female Wistar rat were divided into three groups: control (CT) fed with no HMF; low dose fed with 750 mg/kg/day of HMF and high dose (HD) groups fed with 1500 mg/kg/day of HMF. All groups received these diets for three weeks from postnatal day (PND) 21. The vaginal opening (VO) was monitored daily and euthanasia occurred on PND 44. Gonadotropin, estradiol (E2), progesterone and anti-Müllerian hormone (AMH) concentrations were measured. Reproductive organ weights and ovarian follicle counts were compared.

**Results:**

The HD HMF group had earlier VO. Higher mean luteinising hormone (2.9±1.2 vs 1.3±0.3 mIU/mL) and mean E2 (34.7±8.8 vs 21.2±3.9 pg/mL) and lower mean AMH (2.7±0.5 vs 4.7±0.7 ng/mL) concentrations were found in the HD compared to the CT group. The HD group also had increased number of secondary atrophic follicles.

**Conclusion:**

These results indicate that peripubertal exposure to HMF at HD result in precocious puberty and decreased AMH levels in female Wistar rats.

**What is already known on this topic?**

5-Hydroxymethylfurfural (HMF) is an organic compound that is present at high amounts in processed foods and foodstuffs as a result of heating, roasting, frying and toasting. Data on potential genotoxic, mutagenic, carcinogenic, DNA-damaging, organotoxic and enzyme inhibitory effects of HMF and its metabolites are conflicting. To the best of our knowledge there are no published data about the effects of HMF on pubertal development.

**What this study adds?**

This is the first study of the effects of HMF on pubertal development. The results indicate that peripubertal exposure to HMF in high doses result in precocious puberty and decreased anti-Müllerian hormone levels in female Wistar rats.

## Introduction

5-Hydroxymethylfurfural (HMF) is an organic compound produced by dehydration of fructose and glucose, through a non-enzymatic chemical reaction, in the presence of amino acids (1). The presence of HMF reduces protein digestibility and decreases the nutrition quality of foods. The concentration of HMF is widely used as a parameter to asses honey freshness and appropriate storage conditions (2). It is also ubiquitous in the human diet and is present in high concentrations in processed foods and foodstuffs as a result of heating, roasting, frying and toasting (3). HMF concentration is greater than 1 g/kg in dried fruits, caramel products and some fruit juices and up to 6.2 g/kg in instant coffee (4). It is also present in cigarette smoke, beer and medical products like parenteral solutions containing glucose and pharmaceutical syrups containing fructose (5,6,7,8). Additionally, HMF is used industrially in the production of polymers, surfactants, solvents, pharmaceuticals and plant protection agents (9).

Daily consumption of HMF from diet is estimated to be between 30-150 mg and safe levels of HMF consumption have not been clearly defined (7,10). While the effect of HMF on human health has long been the subject of research, it is not yet clear if HMF represents a potential health risk for humans by dietary exposure. There are conflicting data on potential genotoxic, mutagenic, carcinogenic, DNA-damaging, organotoxic and enzyme inhibitory effects of HMF and its metabolites (11,12,13,14). In terms of carcinogenic effect, HMF derivatives were found to cause hepatocarcinoma and increase skin tumor initiating activity in mice (15). Zhang et al (16) also showed that orally administered HMF in thermolyzed sucrose in rats initiates intestinal aberrant crypt foci formation and causes an increase in both number and size of these lesions in a dose dependent manner. However, in another murine study no evidence of intestinal aberrant crypt formation with HMF or its derivate was reported (17). The US National Toxicology Program (NTP) study of the toxicology and carcinogenesis of HMF in rats and mice, the most comprehensive study on toxic effects of HMF to date, revealed increased incidences of lesions of the olfactory and respiratory epithelium of the nose in rats and mice, and increased incidence of liver cancer in female mice after two years administration of oral HMF. The same study also revealed change in duration of estrous cycles and proportion of regular cycles which may point to possible fertility problems (4). Exposure of children to HMF has increased with changing eating habits in the last decades. No data on the possible toxic effects of HMF on pubertal development has been reported to date. Thus, the aim of this study was to evaluate whether peripubertal exposure to high levels of HMF had any effect on pubertal timing, reproductive organ growth, hormone levels and ovarian follicular development.

## Methods

This study was conducted in Gazi University Laboratory Animal Breeding and Experimental Researches Center (GÜDAM) and approved by Gazi University Local Ethics Committee for animal experiments (approval code: 17.025).

### Animals and Experimental Design

Twenty four Wistar albino rats, weaned on postnatal day (PND) 21, were divided into three equal sized groups (n=8/group). The control (CT) group was given 5 mL/kg/day of tap water, the low dosage (LD) group was given 750 mg/kg day and high dosage (HD) group was given 1500 mg/kg/day of HMF (Sigma 25 mg 5-hydroxymethylfurfural, W501808-25G-K) (4). The treatments were performed orally (gavage), once daily for six days/week, at the same hour (between 9:00 and 10:00 AM), until PND 44. The groups were kept in different cages under identical conditions (22-24 °C, 25-30% humidity, 12 hour light-dark cycle with free access to water and food). Each rat was weighed on PND 21, 26, 33, 40 and 44 just prior to feeding.

### Analysis of Vaginal Opening (VO)

The rats were examined for VO for the assessment of sexual maturity every morning between 9:00 and 10:00 AM. The procedure was performed visually without using a surgical loupe. To compare time of puberty, VO was scored as no VO (0 points), VO between PND 39-44 (1 point) and VO between PND 33-38 (2 points). The scale steps were set by dividing the time period (PND 33-44) that rats had VO into two.

### Euthanasia

The animals were anesthetized by intramuscular xylazine and ketamine (5 and 45 mg/kg, respectively) and then euthanized by cardiac puncture on PND 44, 24 hours after the last dosage of HMF. Blood samples were collected with cardiac puncture on termination day. After centrifugation, serum samples were stored at -80 °C until the time of analysis of follicle stimulating hormone (FSH), luteinizing hormone (LH), estradiol (E2), progesterone (P) and anti-Müllerian hormone (AMH) levels.

### Measurement of Uterus Length, Organ Weight and Assessment of Follicular Score

**Figure 1:**
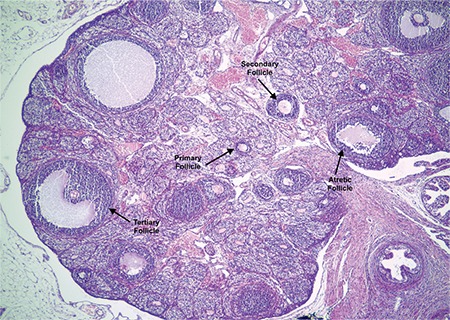
Ovarian photomicrograph from low dosage group showing growth phases of the follicles in x4

After euthanasia, uterus and ovaries were dissected with a limited gross necropsy focused on reproductive organs. Ovaries and uterus were weighed to the nearest 0.001 g with an electric scale (Sartorius Research R200D Electronic Semi-Microbalance). Organ weight per 100 mg of final body weight (relative organ weight) were calculated. In macroscopic analysis, both cervix lengths and uterine corns were measured from fundus to cervix of uterus individually, and the results were recorded. Afterwards, length of the longer corn and the cervical length were added to estimate uterus length. The ovaries and uterus were fixed in 10% buffered formalin, serial sections of 5 µm were made from the mid part of the ovaries and they were stained with haematoxylin and eosin. Four sections were evaluated from each ovary. Follicular quantitative analysis was performed in equidistant sections. Number of follicles at different stages was counted and grouped as healthy secondary, atrophic secondary, healthy tertiary and atrophic tertiary follicles. The follicle was defined as: ‘primary’, if the follicle had one layer of follicular cells; ‘secondary’, if the follicle had two or more layers of follicular cells and was larger than primary follicles; ‘tertiary’, if the follicle had a flood filled antrum and was “atretic”, if the follicle had degenerate oocyte and/or degenerate layers of the membrana granulosa present (18,19). The follicular growth phases are shown in Figure 1. All microscopic analyses were performed with x4, x10, x20, and x40 magnification as a blind test.

### Hormonal Assays

The serum concentration of FSH was determined using a commercial rat-specific enzyme-linked immunosorbent assay (ELISA) kit (Elabscience, E-EL-R0391, Memorial Drive, Suite 216, Houston, Texas, USA) according to the manufacturer’s instructions. The sensitivity of the assay was 1.88 ng/mL. The serum concentration of LH was measured using a commercially available rat-specific ELISA kit (Elabscience, E-EL-R0026, Memorial Drive, Suite 216, Houston, Texas, USA) according to the manufacturer’s instructions. The sensitivity of the assay was 0.94 mlU/mL. The serum concentration of E2 was measured using a commercially available rat-specific ELISA kit (LSBio, LS-F13008, 2401 Fourth Avenue Suite 900, Seattle, WA, USA) according to the manufacturer’s instruction. The sensitivity of the assay was 15.6 pg/mL. The serum concentration of P was measured using a commercially available rat-specific ELISA kit (MyBioSource Inc., MBS762170, San Diego, CA, USA) according to the manufacturer’s instruction. The sensitivity of the assay was <0.188 ng/mL. The serum concentration of AMH was measured using a commercially available rat-specific ELISA kit (Elabscience, E-EL-R0640, Memorial Drive, Suite 216, Houston, Texas, USA) according to the manufacturer’s instruction. The sensitivity of the assay was 0.1 ng/mL. All of these assays were performed concurrently in duplicate and a standard curve was established for assay. Inter‐ and intra‐assay variations were <10%.

### Statistical Analysis

Statistical analysis of the data was performed with Statistical Package for the Social Sciences, version 20 (IBM Inc., Chicago, IL, USA) programme. Values were provided as mean±standard deviation (minimum-maximum). Statistical significance was determined by Kruskal-Wallis one-way analysis of variance for multiple group comparisons and with the Mann-Whitney U test for two-group comparisons. Significance was accepted as p<0.05.

## Results

The study was completed with 23 animals, CT group (n=8), LD group (n=8) and HD group (n=7) as one rat from the HD group died during the experiment from an unknown cause. The mean body weight of the rats was 42.5±1.7 g on PND 21, at the beginning of the experiment. The mean body weight of the CT, LD and HD animals on PND 26, 33, 40 and 44 is given in Table 1. Although mean body weight differed among groups throughout the experiment, the difference was not significant between the groups at the end of the experimental period (PND 44).

Mean age at VO was PND 40±3.2 (range 34-43) in the CT and 35.7±2.7 (range 33-40) in the HD group. Three rats from the LD group did not have VO on termination day. The difference in time of VO was significant (p=0.025). The HD group had VO earlier than both the CT (p=0.023) and LD groups (p=0.018). According to the scale, VO seemed to be slightly delayed in the LD group compared to the CT group, however the difference was not significant (Table 2).

Serum FSH and P concentrations did not differ between the study groups. Serum LH concentrations were significantly higher in the HD group compared to the CT group (p=0.001). However, there was no difference between serum LH concentration in the LD group and the CT group or between the HD group and the LD group. Serum E2 concentrations were increased in the HD group compared to the LD group (p=0.04) and the CT group (p=0.01). Serum AMH concentrations were significantly lower in the HD group compared to both the LD group (p=0.03) and the CT group (p=0.01) (Table 3).

**Figure 2:**
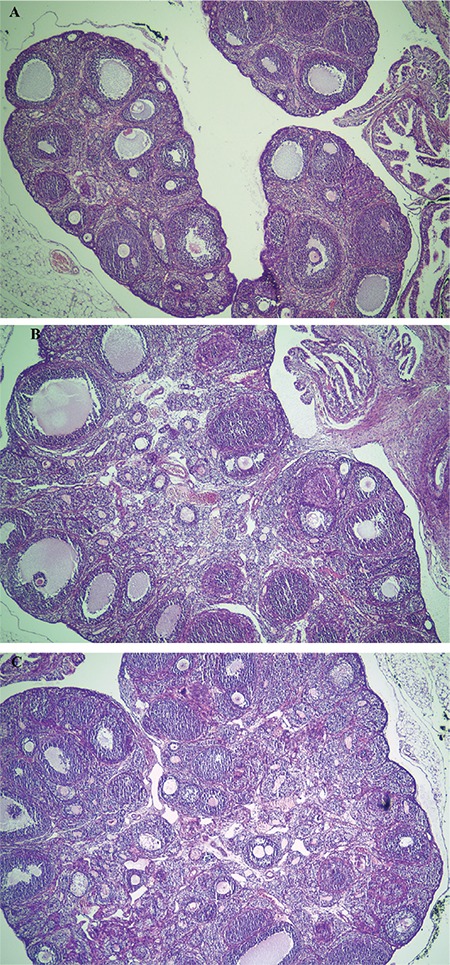
Ovarian photomicrographs of each experimental group in x4 magnitution. (A) Control group (B) low dosage group (C) high dosage group

The mean absolute and relative weight of ovaries and uterus lengths were not different between the groups. The mean absolute and relative uterine weight was increased in the HD group when compared to the CT group (p=0.037 and p=0.005 respectively). The mean number of healthy follicles also did not differ between the groups but the mean number of atrophic secondary follicles was increased in both the LD and HD groups (p=0.02). Measurements of reproductive organs, numbers of follicles and hormone levels are shown in Table 3 and ovarian photomicrographs of each experimental group are shown in Figure 2.

## Discussion

The only study on reproductive and developmental toxicity of HMF was done by the US NTP concerning the toxicology and carcinogenesis of HMF in rats and mice. The study revealed that the duration of the estrous cycle was increased and that regular cycles were fewer in rats that were given 750 mg/kg/day or 1500 mg/kg/day of oral HMF for three months, starting from PND 42. These data indicated the potential of HMF to produce adverse effects in the reproductive system and for fertility (4). In the current study, 750 mg/kg/day or 1500 mg/kg/day of HMF was given orally to female rats starting on PND 21 for three weeks. Rats become sexually mature at the age of six weeks (20). To the best of our knowledge this is the first study investigating the effects of HMF on the reproductive system in sexually immature rats.

Although HMF is mostly present in high calorie foodstuffs, its direct effect on body weight and energy metabolism is controversial. In physiological analyses, redox metabolism is severely affected by HMF, while the effects on the energetics is less well established (21). We found no difference in mean final body weight between the CT and HMF groups. Zaitzev et al (22) reported no change in final body weight of rats receiving 40 mg/kg or 80 mg/kg of HMF for 11 months. The NTP study reported different results for different groups, loss in body weight of rats receiving HMF for three weeks or three months in doses exceeding 750 mg/kg/day and no change in body weight was reported in rats receiving HMF for two years at any dose (4). Heaton and Robinson (23) reported acceleration in body weight gain with 75-225 mg/kg of HMF for an unspecified duration, without giving a detailed description of nutrition conditions. However, it is not appropriate to compare these studies because of the different doses and durations of HMF consumption.

VO, a marker for pubertal onset in rodents, is caused by an apoptotic process in vaginal epithelial cells triggered by increased levels of estrogen. VO of rats of the same strain from different laboratories, or rats of the same strain and laboratory but from different litters, varies hugely. Mean VO time in Wistar rats was reported to range between 33.4±1.98 and 41.6±3.7 days, compatible with the mean VO of the CT group (24). VO in the HD group was also within the reported ranges but it was earlier than in the CT and LD groups. In addition, the E2 concentrations were higher in the HD group, which could be interpreted as high doses of HMF causing precocious puberty in female rats.

Uterine weight increases as puberty progresses in rodents and this increase is associated with E2 levels. However, studies have reported that increased E2 levels my cause a decrease or no change in uterine weight of immature rats (25,26,27). These unexpected results were explained by altered sensitivity of the estrogen receptors in the uterus due to high E2 concentrations or due to the substance used in the experiment (27). In this present study, absolute and relative uterine weight was increased in the HD group and both LH and E2 concentrations were higher in the HD group compared to the CT and LD groups. It may be that HMF somehow activated the hypothalamo-pituitary system resulting in increased E2 concentrations, led to early VO and may also have caused an uterotrophic effect. Detailed physiological studies are needed to understand and explain the mechanism fully.

Intense maturational changes in the hypothalamic-pituitary system are accompanied by an increase in gonadotropin response in the ovaries, resulting in development of gonadotropin related follicles. Measuring ovarian weight and microscopic examination are indispensable steps for female reproductive toxicology studies (28). In our study, absolute or relative ovarian weight did not vary between groups but number of atrophic secondary follicles was increased in the HMF groups compared to the CT group. Numerous atrophic follicles may be present at the normal peripubertal stage, before ovulation. As the rat matures and cyclicity is set after several cycles, the number of atrophic follicles decreases. Atrophic follicles are prominent in rats that are euthanized around PND 42 (28). However, ovarian toxicology studies have shown that an increased number of atrophic follicles was among the most common histopathologic features indicating ovarian detriment, even in rats at six weeks of age (29). As 3/8 LD rats did not have VO on necropsy day and 2/8 had VO the day before necropsy, an increased number of atrophic follicles in the LD group may be attributed to their immaturity, but the ovaries of the HD group seem to have been affected by HMF toxicity.

AMH is produced by growing ovarian follicles and reflects the antral follicle count (30). Rodent studies have shown that AMH has a critical role in initial follicle recruitment and selection of dominant follicles (31). Decrease in serum AMH correlates directly with the decrease in the number of growing follicles (32). In this study, AMH concentrations were significantly decreased in the HD group, which may indicate decreased ovarian reserve and HMF-related ovarian damage. Although the role of E2 in AMH expression is not clear, another possible cause for the decline in AMH concentrations may be increased concentrations of E2. Increased E2 has been shown to reduce the activation of AMH promoter in some *in vitro* studies (33,34,35). In contrast there are also studies supporting the opposite or showing that E2 has no direct effect on AMH (36,37,38). Thus, the relationship between AMH and E2 concentrations is still controversial.

## Conclusion

HMF is present in numerous foodstuffs at high levels and peripubertal children have an increasing exposure to this potentially toxic metabolite with changing dietary habits. This is the first study of the toxic effects of HMF in peripubertal rats and it was shown that high doses of HMF given orally for three weeks caused early VO, an increased number of secondary atrophic follicles and decreased AMH concentrations. However, these results may not be directly related to humans given the experimental dosage and duration applied in this rat model. Therefore, there is a need for further studies to elucidate the mechanisms leading to these findings.

## Figures and Tables

**Table 1:**
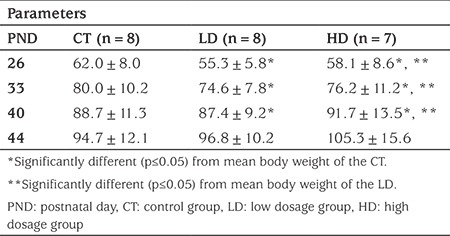
Mean body weight (g) of each experimental group on different postnatal days

**Table 2:**
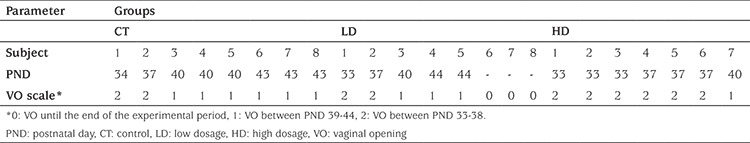
Vaginal opening time (in days) in different experimental groups

**Table 3:**
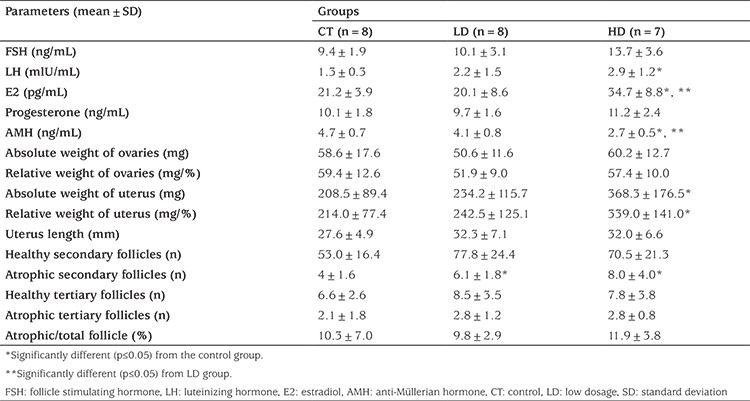
Measurements of mean serum hormone concentrations, weight/length of reproductive organs and follicle counts of the study groups
